# Evaluation of the Efficacy of a Lactobacilli-Based Teat Detergents for the Microbiota of Cows Teats Using an Untargeted Metabolomics Approach

**DOI:** 10.4014/jmb.2305.05016

**Published:** 2023-09-22

**Authors:** Rui Yan, Zhongqing Ji, Jiaqi Fan, Jiang Li, Yan Ren

**Affiliations:** 1 Inner Mongolia Key Laboratory for Biomass-Energy Conversion, Inner Mongolia University of Science and Technology, Baotou 014010, P.R. China; 2 School of Life Science and Technology, Inner Mongolia University of Science and Technology, Baotou, 014010, P.R. China

**Keywords:** *Lactobacillus plantarum*, subclinical mastitis, teat detergents, metabolomics

## Abstract

Teat cleaning pre- and post-milking is important for the overall health and hygiene of dairy cows. The purpose of this research was to evaluate the effectiveness of a teat detergents based on lactic acid bacteria according to changes in somatic cell count and cow-milk metabolites. Sixty-nine raw milk samples were collected from 11 Holstein-Friesian cows in China during 12 days of teat cleaning. An ultra-performance liquid chromatography-quadrupole-time of flight mass spectrometry-based untargeted metabolomic approach was applied to detect metabolomic differences after treatment with lactic acid bacteria and chemical teat detergents in cows with subclinical mastitis. The results suggest that the lactobacilli-based teat detergents could reduce somatic cell count and improve microhabitat of cow teat apex by adjusting the composition of metabolites. Furthermore, the somatic cell count could be decreased significantly within 10 days following the cleaning protocol. Lactic acid bacteria have the potential to be applied as a substitution to teat chemical detergents before and after milking for maintenance of healthy teats and breasts. Further, larger scale validation work is required to support the findings of the current study.

## Introduction

Subclinical mastitis (SCM) is one of the highly infectious diseases in dairy cows with the characteristics of high incidence and nonvisible clinical symptoms [1]. Presently, SCM is diagnosed according to the somatic cell count (SCC) and the California mastitis test (CMT). The latter gives a positive result only when the SCC is higher than 400,000 cells/ml, but infection is considered present when the SCC is higher than 200,000 cells/ml. A cutoff of 200,000 cells/ml has been applied for identifying SCM. In addition, it has been reported that an SCC of less than 100,000 cells/ml is confirmed to be a healthy level of somatic cells [2].

In China, teat detergents are usually used to control cow mastitis in the pasture. Teat cleaning before and after milking is vital for decreasing the risk of cow mastitis and reducing the possibility of microbial contamination of raw milk [3]. At present, various teat-dipping detergents, including phenolic compounds, chlorine, chlorhexidine, sodium hypochlorite, iodine-based gel, iodophor solution, and alcohol, are applied for pre-milking teat dipping [4]. Although these chemical detergents can prevent pathogen infections, the potential chemical residues in raw milk were created by the high concentration of these chemical matter [5, 6]. As a consequence, previous researchers have been focused on developing natural substances such as plant extracts as novel teat detergents to decline microbial contamination in raw milk [7].

Currently, lactic acid bacteria (LAB) are regarded as the best choice for controlling various infectious diseases such as human and bovine mastitis [8, 9]. As we know that LAB are safe, non-pathogenic, and possess lots of potential traits which can be applied to prevent human or bovine mastitis [10, 11]. John *et al*. [12] developed a liquid product containing a mixture of *Lactobacillus* organisms as a post-milking teat spray. In our previous study, we used a novel lactobacillus-based teat disinfectant for improving bacterial communities in the milks of cow teats with subclinical mastitis [8]. Therefore, we hypothesized that LAB would be at least as effective as a commercially available iodine-based post-milking teat disinfectant in improving udder health. In our study, two *Lactobacillus* (L.) *plantarum* strains (IMAU10155 and IMAU80065) were screened from 347 *L. plantarum* strains (from the LAB Culture Collection at Inner Mongolia Agricultural University) were isolated from traditional fermented dairy products and pickles in China. Both of two *L. plantarum* strains can generate bacteriocins which exhibit inhibitory activity against pathogenic bacterium, such as *Escherichia coli* and *Staphylococcus* [13]. This potential prompted us to examine the applicability of ultra-performance liquid chromatography-quadrupole-time of flight mass spectrometry (UPLC-MS)-based untargeted metabolomics for identifying metabolite changes occurring in the milk of cows suffering from SCM after treatment with chemical teat detergents (CD) and LAB teat-dipping, an approach that has not been documented thus far. Thus, the aim of this research was to evaluate the efficacy of the LAB teat detergents and chemical teat detergents by UPLC-MS and, furthermore, to determine if there was any difference in the common metabolites or SCC among the milk of SCM dairy cows reared on the same farm. This is the first reported method for detecting the metabolites of cow teats’ milk after cleaned by LAB teat detergents, which may help shedding new light on improving the microbiota of cow’s teats.

## Materials and Methods

### Raw Milk Collection and Determination of SCC

This study used 25 lactating Holsteins dairy cows from sheds of Mengele pasture of Inner Mongolia. All of the dairy cows which kept in a tied housing system were 2 to 4 years, fed with total mixed rations (hay 5 kg/d, corn silage 25 kg/d, mixed concentrate 10 kg/d, sodium bicarbonate 0.15 kg/d), and milked with an automatic milking system. Members of the research team collected milk samples during the morning milking. Before the experiment, researchers firstly washed the cow udder with sterile water and then wiped them with disposable cloths for primary mammary sanitation. Subsequently, researchers removed several streams of foremilk before sample collection. One hundred milliliter cow milk samples were collected in a sterile tube for detection of SCC using a laser beam Bentley FTS/FCM400 Combi Instrument (USA). According to the criteria of the cutoff of 200,000 cells/ml for distinguishing SCM, 11 cows were diagnosed with SCM in our study. All researchers used sterile gloves during the sampling process.

Following the primary mammary sanitation, we separated the left and right teats into two groups. The left two teats were treated with LAB teat detergents containing 5 × 10^10^ colony-forming units/ml *L. plantarum* (LAB group), which proved the optimal viable counts concentration by our previous studies [8, 13]. The right two teats were treated with the CD (Dipal Concentrate 1+4, Delaval, Tianjin) as the control group (CD group). In total, 100 ml milk were collected from two quarters (50 ml milk from one quarter) which were blended within group for subsequent analysis. The sampling time points were 0 day (before treatment), 1 day, 10 days, and 12 days (after 10 days of continued treatment, five cows were selected randomly from each group to carry on cleaning 2 days with sterile water which replaced the LAB and CD). In total, 76 milk samples from 11 SCM cows were kept on ice and then immediately moved to our laboratory. There were 7 milk samples deleted from following analysis for the possibility of contamination of cow feces.

### UPLC-Q-TOF MS and MS^E^ Analysis

**Reagents and Chemicals**

For Q-TOF analysis, HPLC-grade water was obtained by purifying demineralized water in a Milli-Q plus system (Millipore, USA). Optima LC/MS-grade acetonitrile, ammonium hydroxide (> 99%), and formic acid (98–100%) were acquired from Sigma Aldrich (USA). Leucine-enkephalin, applied as the lock mass, was purchased from Waters/Micromass (UK).

### Sample Treatment and Injection

Prior to UPLC-Q-TOF, the 69 skimmed milk samples were thawed and thoroughly vortex mixed. A 2 ml aliquot of each milk sample was added to 14 ml of acetonitrile and mixed by vortexing for 2 min and ultrasonic processing for 5 min. Next, the sample was centrifuged at 12,000 ×*g* for 15 min at 4°C to remove large biomolecules, such as proteins. The supernatant was then vacuum concentrated and dissolved in 500 μl 40% (v/v) acetonitrile. Before transferring to autosampler vials, the samples were filtered through a 0.22 μm MS nylon syringe filter (Sigma-Aldrich).

### Xevo G_2_ QTOF

An Acquity UPLC system (Waters, USA) was interfaced with a Xevo G_2_ QTOF mass spectrometer (Waters/Micromass) using an orthogonal Z-spray electrospray ionization (ESI) interface. Chromatographic separation was performed using an Acquity UPLC BEH T3 column (1.8 μm; 2.1 × 100 mm) from Waters, using a flow rate of 0.3 ml/min. The mobile phases used for positive ion acquisition mode, (A) ddH_2_O and 0.1% formic acid and (B) acetonitrile and 0.1% formic acid, were increased linearly as follows: 1 min, 95%; 6 min, 60%; 18 min, 15%; 18.5 min, 10%; 22 min, 10%; 22.50 min, 95%, and 25 min, 95%. The total run time was 25 min. The mobile phases used for negative ion acquisition mode were (A) ddH_2_O and 0.1% ammonium hydroxide and (B) acetonitrile. The mobile phases for gradient elution were the same as in positive ion acquisition mode. The injection volume was 10 μl. Nitrogen (Praxair, Spain) was applied as both the drying and nebulizing gas. The desolvation gas flow rate was set at 600 L/h. The resolution of the TOF mass spectrometer was ~22,000 with full width half maximum at m/z 556.2771; MS data were obtained over an m/z range of 50–1000 with a scan time of 0.7 s. A capillary voltage of 3.0 kV and 2.5 kV was used in positive and negative ionization mode, respectively. A cone voltage of 40 V was applied. Argon was used as a collision gas (99.995%; Praxair). The desolvation temperature was set to 350°C, and the source temperature was 100°C. The column temperature was set to 35°C, and the autosampler was at room temperature. The MS and MS^E^ spectra of the cow milk metabolites were obtained using a collision energy ramp from 20 to 60 eV. The accurate masses and compositions of the precursor and fragment ions were calculated and sequenced using MassLynx software (Waters).

Quality-control (QC) samples were prepared by mixing sample extracts to analyze the repeatability of samples under the same processing method. During the analysis of the instrument, a QC sample was inserted into every 5 detection and analysis samples to monitor the repeatability of the analysis process.

### Data Processing and Statistical Analysis

All of the MS raw data were processed statistically using MetaboAnalyst software. Metabolites that were frequently (> 50%) below the limit of detection or had at least 50% missing values were deleted from consideration. Data normalization of metabolite concentration was performed to create a Gaussian distribution prior to statistical analysis and pathway analysis [14]. In this research, we performed log transformation and auto-scaling of metabolite values. The different metabolites in MS^E^ mode were identified using Progenesis QI software based on m/z, retention time, and fragment information.

Univariate analysis of continuous data was performed using fold change analysis and *t*-tests (rank sum) done in MetaboAnalyst and Progenesis QI (Nonlinear Dynamics, UK) [15]. To perform a standard cross-sectional two-group study, we compared the CD group with the LAB group at each time point (0 d, 1 d, 10 d, and 12 d) separately by one-way analysis of variety (ANOVA). Multivariate analysis, such as principle component analysis (PCA) and orthogonal partial least squares–discriminant analysis (OPLS-DA), is showed between different times (0 d, 1 d, 10 d, and 12 d) in the LAB and CD teat dipping groups. PCA and OPLS-DA were performed using MetaboAnalyst and Progenesis QI. Three criteria were used for defining measuring differences in metabolites between groups: minimum fold change (FC) (≥ 2), *t*-test *p*-value (≤ 0.05), and variable importance (VIP) (≥ 1). Qualifying filtered variables were further analyzed and identified.

Biomarker profiles and the quality of the biomarker sets were determined using receiver-operator characteristic (ROC) curves as calculated by MetaboAnalyst 4.0. Paired sensitivity and false-positive rations (1-specificity) at different classification decision boundaries were calculated. A ROC curve is plotted with sensitivity values on the Y-axis and the corresponding false-positive rates (1-specificity) on the X-axis. ROC curves are often summarized into a single metric known as the area under the ROC curve (AUC), which indicates the accuracy of a test for correctly distinguishing one group such as LAB_1 from LAB_10.

### Identification of Metabolites and Metabolic Pathway Analysis

Fragmentation patterns, obtained in MS^E^ and MS/MS modes in Q-TOF-MS, were applied for structural evaluation of marker features. Where possible, marker identification was confirmed by matching fragmentation patterns to authentic standards. Subsequently, database searches were done. Database searches were performed in HMDB (Human Metabolome Database; www.hmdb.ca), MassBank (www.massbank.jp), MMCD (Madison Metabolomics Consortium Database; http://mmcd.nmrfam.wisc.edu/), and Metlin (http://metalin.scripps.edu/). The differential metabolites were further analyzed using the MetaboAnalyst 4.0 web server (http://www.metaboanalyst.ca/) combined with the KEGG PATHWAY database (http://www.kegg.jp/kegg/pathway.html) [15].

## Results

### Determination of Somatic Cell Count

**Fig. 1:**
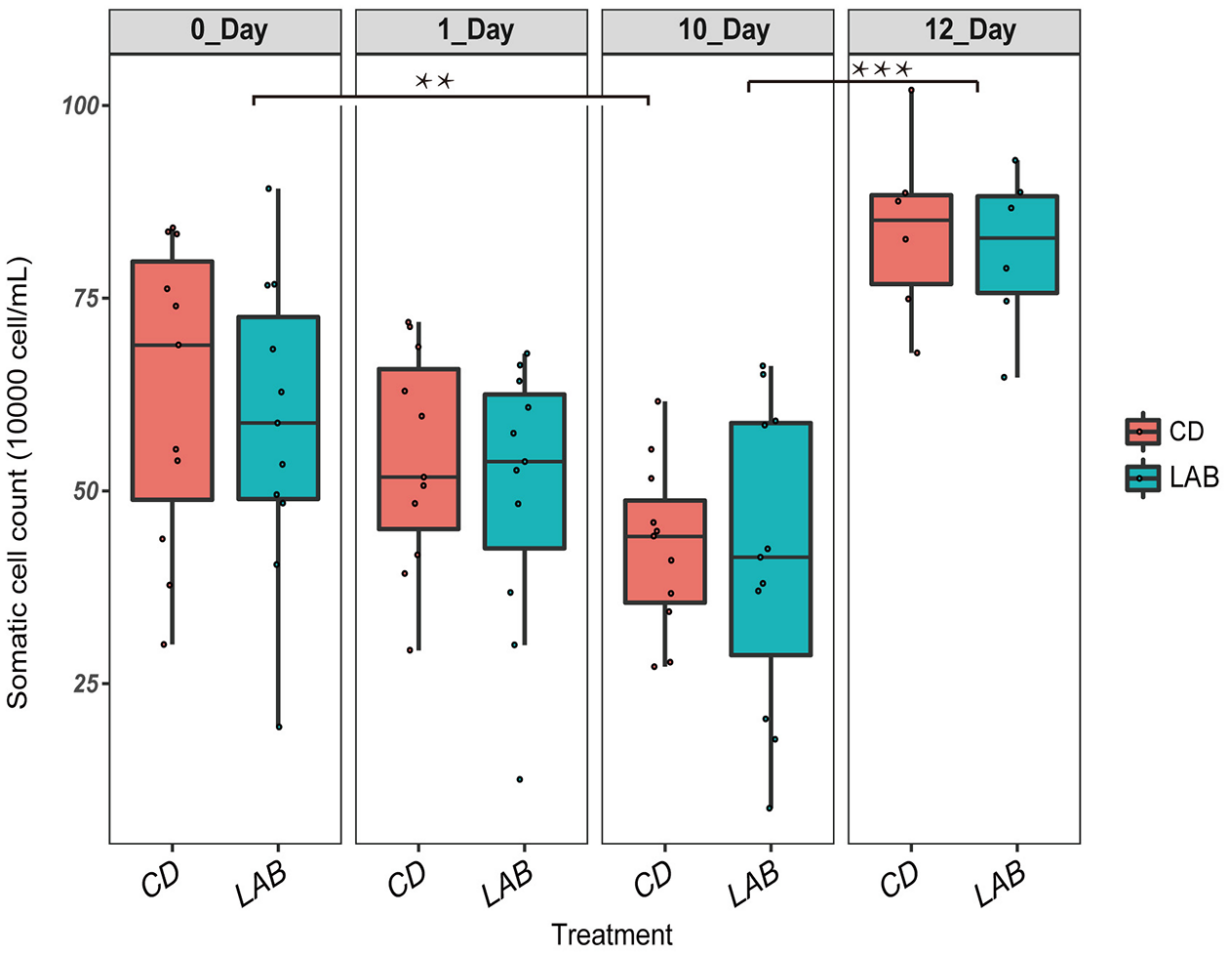
The changes of SCC in cow milk during cleaning process with LAB and CD. Significant differences between sample pairs were evaluated by pairwise Mann-Whitney test with Bonferroni correction; *p < 0.05, ** *p* < 0.01, and *** *p* < 0.001.

The SCC of 69 milk samples shown in Fig. 1 scaled from 6.7 × 10^4^ to 1.02 × 10^6^ cells/ml. It was observed that the SCC in both LAB and CD groups declined gradually during the cleaning process. In addition, the SCC of LAB group was marginally lower than that of CD group, while after using sterile water instead of LAB and CD, the SCC ascended rapidly.

### Metabolomics Data Analysis

Metabolomic data were obtained from 11 SCM cows at four different time points: 0, 1, 10 and 12 days. A total of 2,005 metabolites (1,240 and 765 in positive and negative ion mode, respectively) were detected from among all 69 samples.

### Comparison of Metabolite Composition Following Cleaning between LAB and CD Detergents

**Fig. 2:**
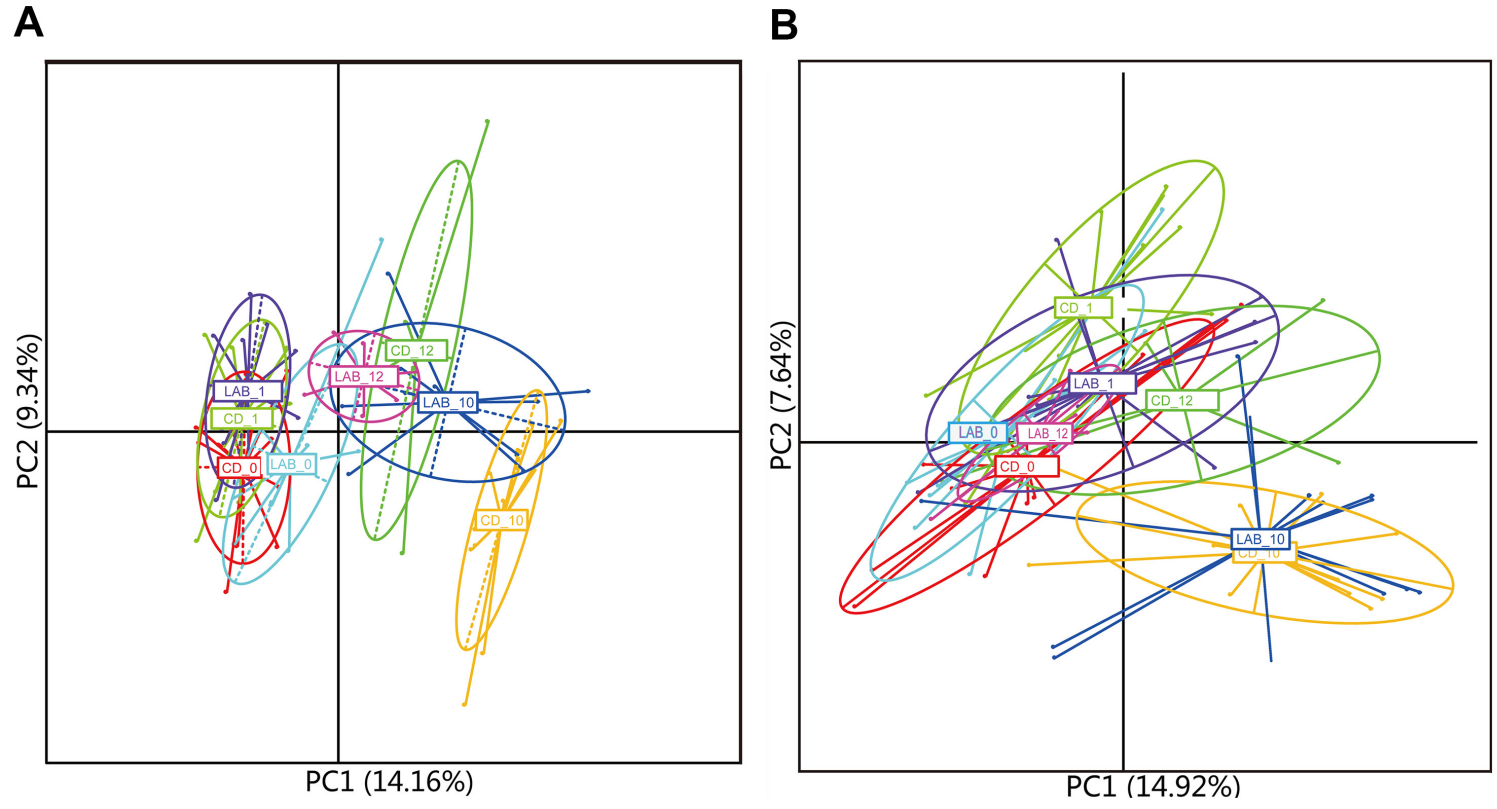
PCA score plots of 8 milk groups in positive (A) and negative (B) ion modes. Each symbol represents the cow milk metabolites of one sample; sample group is represented by the respective color.

To visualize the metabolic differences among the eight milk groups (CD_0, CD_1, CD_10, CD_12, and LAB_0, LAB_1, LAB_10, LAB_12), an unsupervised PCA was performed based on the metabolome generated in the positive and negative modes (Fig. 2). Both PCA score plots (PC1 and PC2 accounted for 14.16% and 9.34% of the total variance, respectively [Fig. 2A] and (PC1 and PC2 accounted for 14.92% and 7.64% of the total variance, respectively [Fig. 2B]) revealed apparent differences in metabolite profile, as the symbols representing the samples of the LAB groups and CD groups at 0 d and 10 d were separated with only minor overlap on both positive and negative score plots. Nevertheless, the symbols representing the milk samples of the other six groups overlapped.

**Fig. 3:**
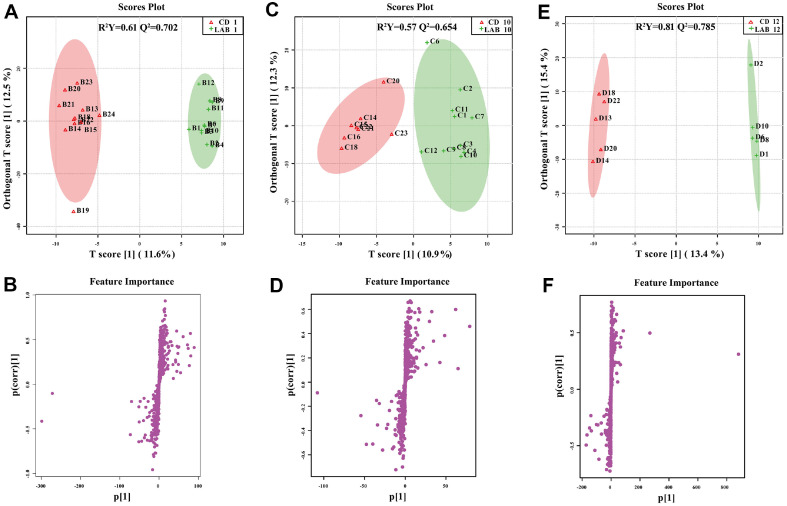
OPLS-DA and S-plots showing pairwise comparison between LAB and CD groups from different time-point in positive ion mode. R^2^Y and Q^2^ are fitness power and predicting power, respectively. On the score plots, sample group is represented by the respective color; on the S-plot, each dot corresponds to a single metabolite.

**Fig. 4:**
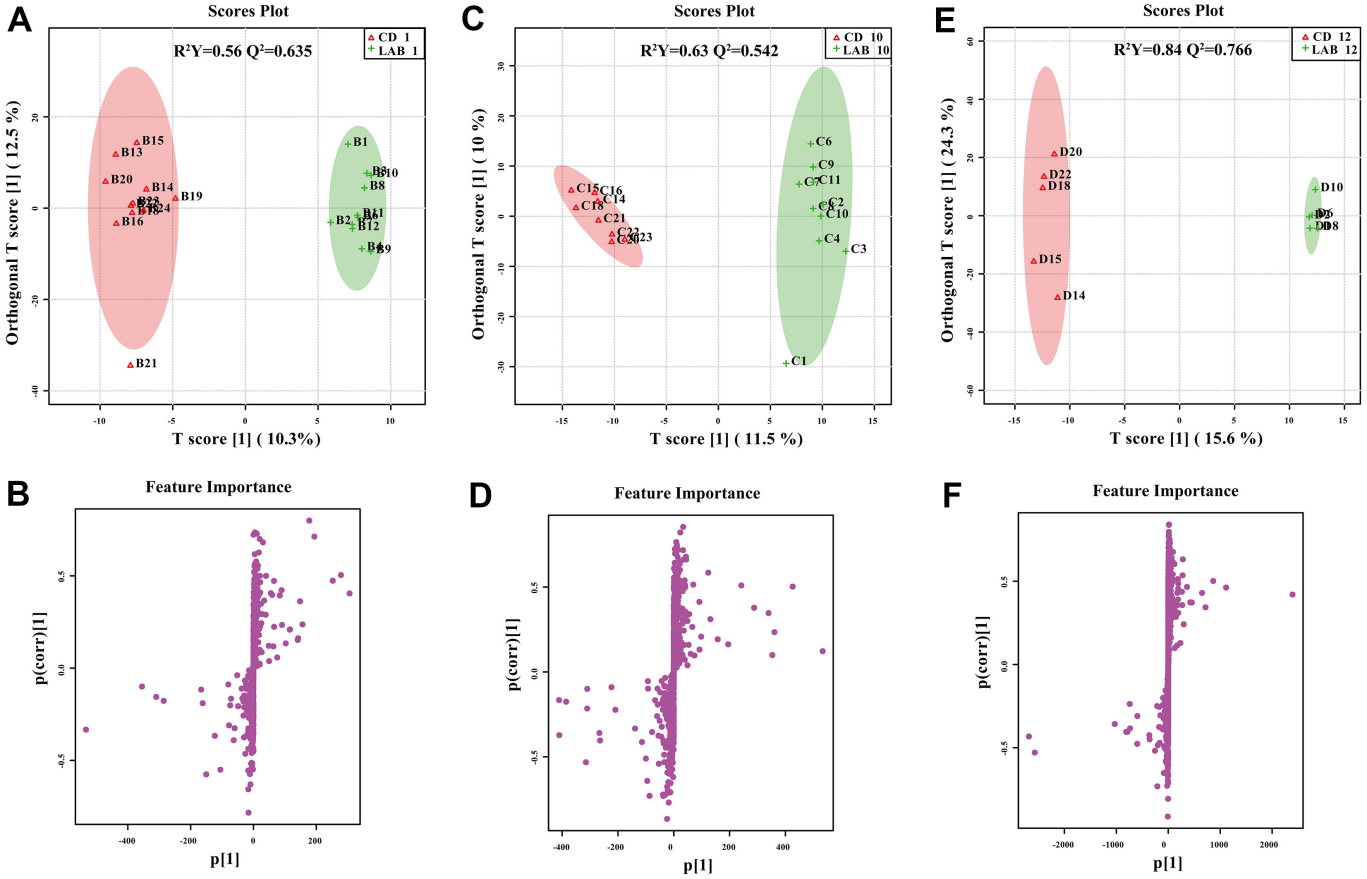
OPLS-DA and S-plots showing pairwise comparison between LAB and CD from different time-point in negative ion mode. R^2^Y and Q^2^ are fitness power and predicting power, respectively. On the score plots, sample group is represented by the respective color; on the S-plot, each dot corresponds to a single metabolite.

It is noticeable that there is difference between the LAB group and the CD group at the different time points. Using one-way ANOVA test and supervised OPLS-DA, we compared efficacy between the CD group and LAB group at three time points separately. The score plots and S-plots in the positive and negative ion modes are shown in Fig. 3 and Fig. 4. The R^2^Y and Q^2^ values were above 0.56 and 0.542, respectively, indicating that significant difference between the groups cleaned with the LAB and CD detergents. In the S-plots, each dot represents a variable; with greater distance from the origin, the magnitude of each variable’s contribution to the group difference is greater.

Lists of 21 significantly regulated metabolites (Table 1) were selected for each comparison using a threshold value for absolute FC (≥2), ANOVA *P*-value (≤0.05), and VIP (≥1). Compared with the CD groups, there were 3 (up), 12 (6 up and 6 down), and 6 (5 up and 1 down) putative metabolites differentially expressed at 1 d, 10 d, and 12 d in the LAB groups, respectively. Detail information of the metabolites is shown in Table S1.

### Changes in Metabolite Composition Following Cleaning by LAB Detergents over Time

**Fig. 5:**
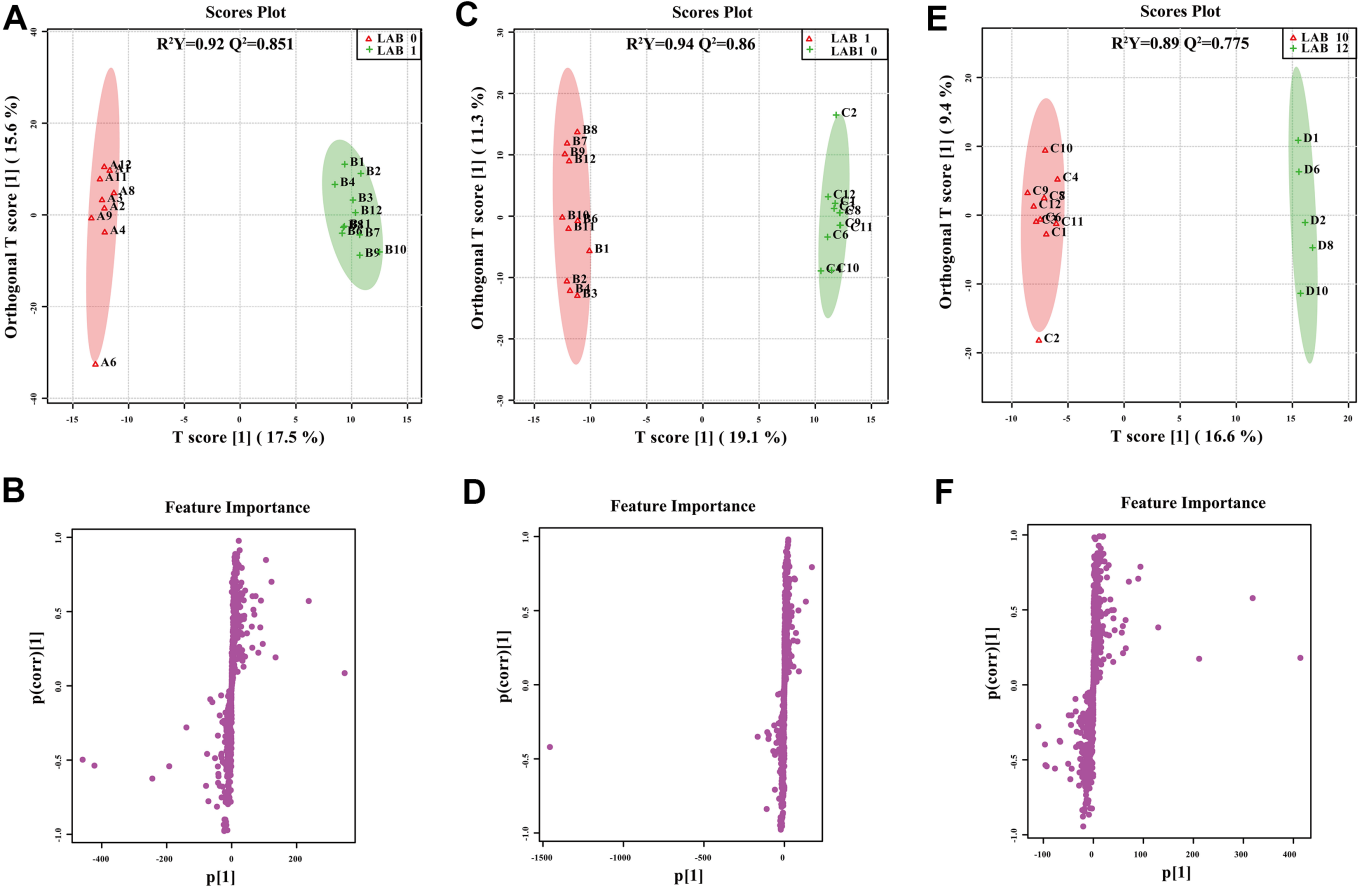
OPLS-DA and S-plots showing pairwise comparison between LAB groups from different timepoint in positive ion mode. R^2^Y and Q^2^ are fitness power and predicting power, respectively. On the score plots, sample group is represented by the respective color; on the S-plot, each dot corresponds to a single metabolite.

**Fig. 6:**
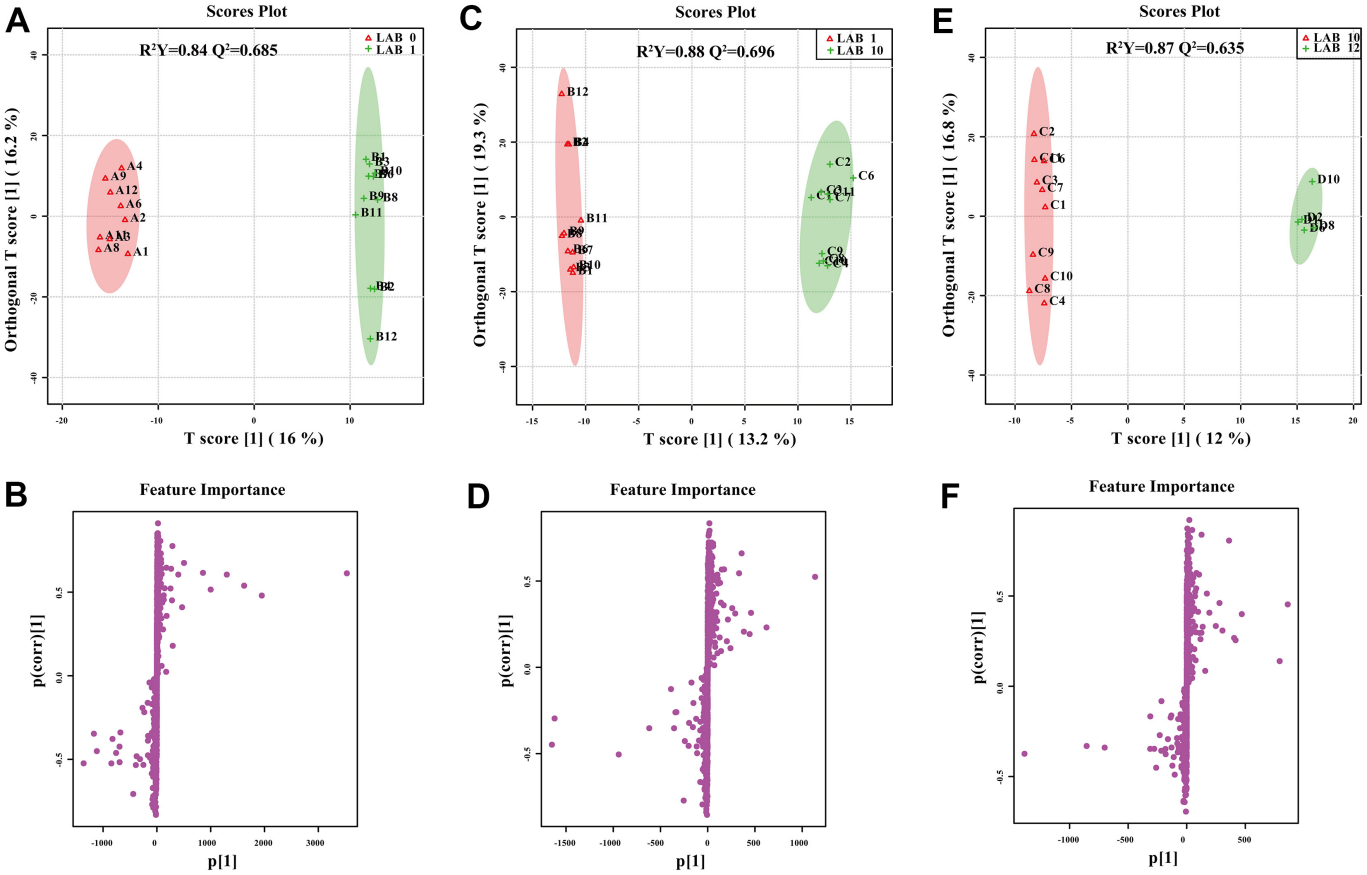
OPLS-DA and S-plots showing pairwise comparison between LAB groups from different timepoint in negative ion mode. R^2^Y and Q^2^ are fitness power and predicting power, respectively. On the score plots, sample group is represented by the respective color; on the S-plot, each dot corresponds to a single metabolite.

The data were further processed by a supervised OPLS-DA and one-way ANOVA test to identify differences in metabolites among the LAB groups at the four time points. The score plots and S-plots in both positive and negative ion modes are shown in Fig. 5 and Fig. 6. The R^2^Y and Q^2^ values were above 0.84 and 0.635, respectively, for all six models, indicating high fitness and predictive value. The top 15 most important metabolites that separated the two groups are shown in the VIP plots (Figs. S1 and S2), Top 3 metabolites with greater VIP score were used for ROC curve analysis. The Areas Under Curve (AUC) of all groups were more than 0.8 that indicated these biomarkers have very good predictive abilities. Lists of significantly regulated metabolites (Table 2) were selected for each comparison using the threshold of an absolute FC (≥ 2), ANOVA *p*-value (≤ 0.05), and VIP value (≥ 1). Compared with the pre-cleaning time point, there were 35 (23 up and 12 down), 34 (30 up and 4 down), and 22 (19 up and 3 down) differentially expressed putative metabolites at the 1 d, 10 d, and 12 d post-cleaning, respectively. Detail information of the metabolites is shown in Table S2.

### Metabolic Pathway Analysis

MetaboAnalyst 3.0 was used to investigate and map metabolic differences among LAB_0, LAB_1, LAB_10, and LAB_12, as reflected by significant differences in milk metabolites. The major metabolic pathways are those directly involved in carbohydrate, energy, vitamin, lipid, peptide and amino acid metabolism.

**Fig. 7:**
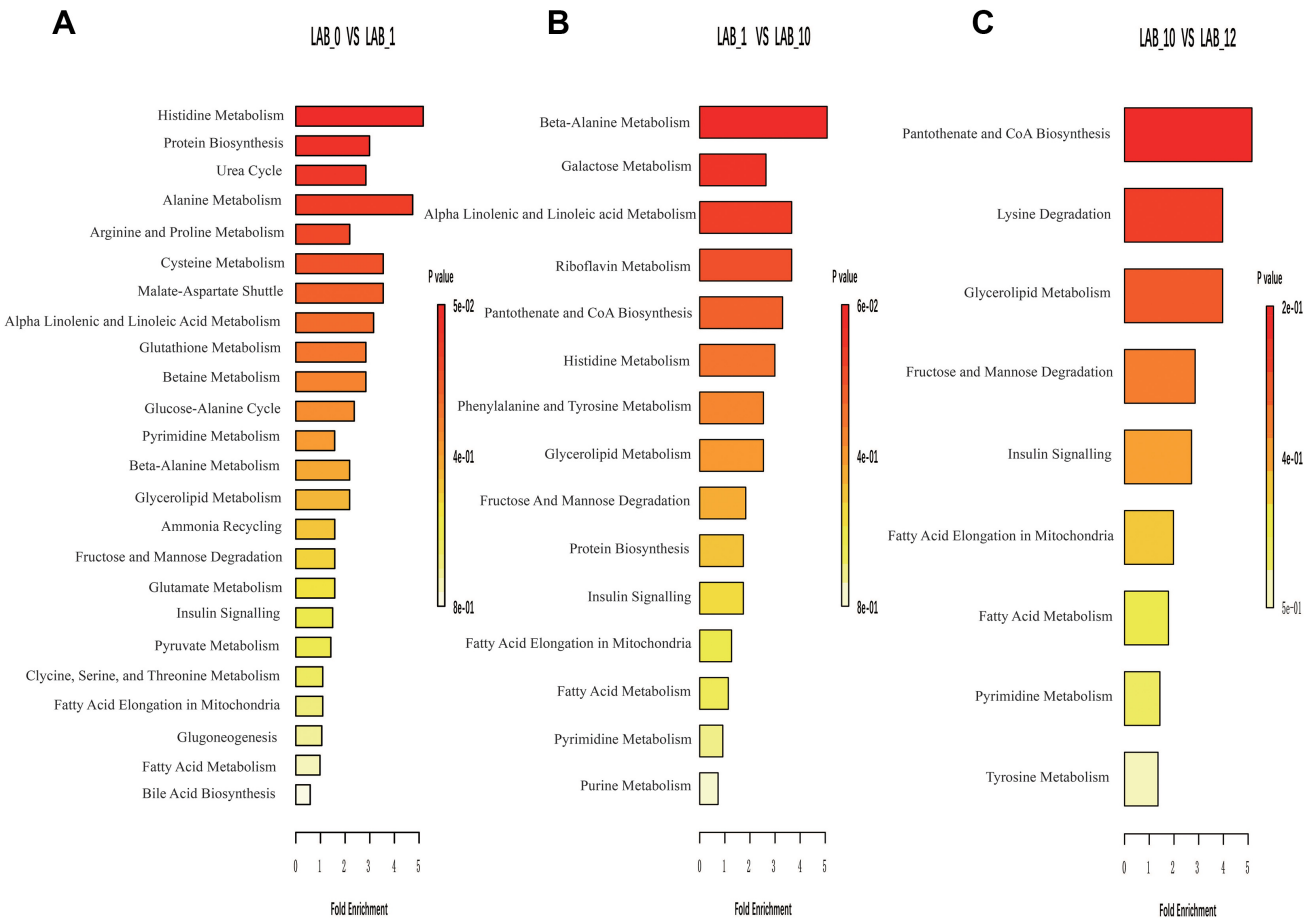
Summary plots for quantitative enrichment analysis between (A) LAB_0 and LAB_1 groups, (B) LAB_1 and LAB_10 groups and (C) LAB_10 and LAB_12 groups.

Metabolite enrichment analysis showed that histidine metabolism (carnosine and inosine), protein biosynthesis (L-glutamate, L-threonine, and hippurate), the urea cycle (aspartate and urea), alanine metabolism (L-glutamate), and arginine and proline metabolism (L-glutamate and urea) were the five most enriched pathways in the LAB_0 and LAB_1 groups (Fig. 7A). In the LAB_1 and LAB_10 groups, beta-alanine metabolism (carnosine and pantothenic acid), galactose metabolism (D-galactose, alpha-lactose, and galactinol), alpha linolenic acid and linoleic acid metabolism (linoleic acid and arachidonic acid), riboflavin metabolism (riboflavin), and pantothenate and CoA biosynthesis (pantothenic acid, CoA, and pantetheine) were the five most enriched pathways (Fig. 7B). In the LAB_10 and LAB_12 groups, pathway enrichment analysis showed that pantothenate and CoA biosynthesis (pantetheine), lysine degradation (allysine), glycerolipid metabolism (palmitic acid, stearic acid, and oleic acid), fructose and mannose degradation (fructose-6-phosphate), and insulin signaling (palmitic acid) were the five most enriched pathways (Fig. 7C).

## Discussion

Bovine mastitis has received wide attention because it causes major economic losses within the dairy industry. It was well known that many diseases were caused by the existence of pathogenic microbes in raw milk [16]. Teat cleaning before and after milking is recommended to reduce bacterial load in raw milk [8, 17]. Hence, it is necessary to develop a novel teat detergent to chemical detergents. Probiotic LAB exhibited the ability of inhibiting pathogenic microbes and improving the intestinal microflora and environment [8, 10]. In particular, some probiotic LAB has been reported to be capable of curing and preventing dairy cow mastitis when fed directly, thus representing a potential alternative to antibiotic strategies [10].

To the best of our knowledge, our study is the first to describe the bovine teat metabolite profiles associated with LAB disinfection via UPLC-MS. Hence, our findings provide new insights into whether probiotic LAB regulates the metabolite composition of the teat and suggests a role for LAB in the prevention of SCM. Additionally, our data confirm the efficacy of LAB disinfectant for reducing the SCC of raw milk during the cleaning process.

At present, the SCC is used to distinguish cow mastitis, as it naturally exists in raw milk. In this experiment, the SCC gradually declined during the course of teat cleaning treatment. According to Fig. 1, the SCC of cleaning with LAB was marginally lower than that of the CD group. However, after two days of washing cows’ teats with sterile water instead of the two cleaning agents, SCC in milk increased significantly, further indicating that both LAB and CD can reduce SCC in milk from cows' teats. Although the SCC did not reach to a healthy level, changes of metabolites of raw milk is associated with the SCC.

According to the multivariate statistical analysis comparing the LAB groups and the CD groups, there was significant difference in the metabolites of the milk samples after cleaning by LAB and CD. Together with the change of SCC seen with both the LAB and CD cleaning processes, as shown in Fig. 1, we can deduce that LAB cleaning has the same efficacy as CD cleaning for preventing SCM. However, several metabolic differences were still observed in the milk samples collected from the LAB group cows compared with the CD group samples at each time point (Tables 1 and 2).

**Fig. 8:**
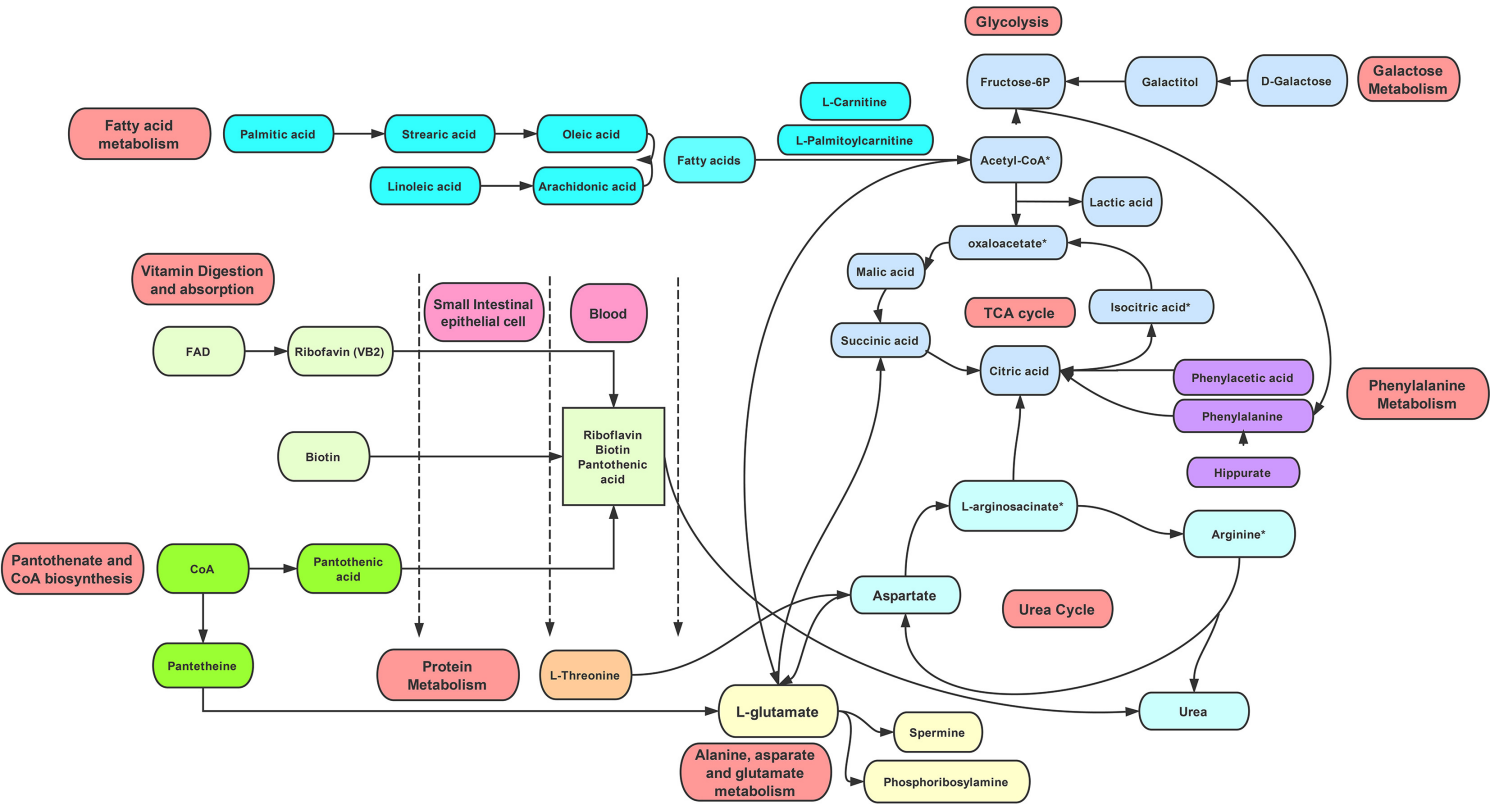
Schematic overview of changes in the metabolite pathway effected by LAB disinfectant. Sections with different colors represent different metabolic pathway. * represents undetected metabolites.

Metabolites of vitamins were significantly different between milk from the LAB group and the CD group at three time points (Table 1). The trend of vitamins (riboflavin (VB_2_), cis-9-retinal, and biotin (VH)) from the LAB group to the CD group is down-regulated in 1-day and 10-day milk samples, respectively; meanwhile, lactic acid showed a trend toward up-regulation from 0 to 1 days after LAB teat dipping treatment. It was previously reported that this group of metabolites, including VB_2_, cis-9-retinal, and VH, can enhance lactic acid production during fermentation by *L. paracasei*, and that lactic acid can be applied to control the growth of pathogenic bacteria including *E. coil* and *S. aureus* [18]. This convincingly demonstrated that LAB detergent could be transferred through milk ducts into the teat and mammary gland, thus further regulating bacterial metabolites of the teat. Furthermore, we observed an increased abundance in almost all small peptides (less than or equal to 4 amino acid residues in length) in milk samples from CD and LAB groups over the time course. It has been proposed that small peptides are associated with milk somatic cells, because the milk SCC correlates with both protease release (and subsequent proteolysis activity) and the severity of mastitis. As shown by multivariate analysis of the metabolomics data of the LAB groups, a noticeable finding of this research is the change in metabolite composition of cow milk over the course of LAB teat dipping treatment (Fig. 8). Regarding vitamin digestion and absorption, VB_2_, VH, and pantothenic acid (VB_5_) were detected and found to be related to pantothenate and CoA biosynthesis. From 0 to 10 d with the LAB teat dipping treatment, VB_2_ showed a trend toward up-regulation, but a trend toward down-regulation was seen between 10 and 12 d. In previous research, VB_2_ was shown to be converted within the body to flavin adenine dinucleotide (FAD), which is known to act physiologically as a coenzyme for redox reactions. As FAD is capable of acting physiologically as a coenzyme for redox reactions. Then the neutrophil function is activated, because VB_2_ injection increased or regulated cytokine production [19]. Another study done with Indian water buffaloes and showed that supplementation with vitamins A and H can help ameliorate the altered milk oxidant/antioxidant balance and thus ensure recovery from SCM [20].

Activity in the glycolysis, galactose metabolism, and citrate cycle (tricarboxylic acid [TCA] cycle) pathways was associated with the metabolism of certain amino acids, such as phenylamine, as well as with fatty acid metabolism and the urea cycle. During the LAB teat dipping treatment, a group of carbohydrates including glucose, fructose-6-phosphate, D-galactose, and galactitol showed an increased concentration, but after cessation of teat dipping, the concentration of glucose was decreased. As mastitis progresses, pathogens might use up more of these compounds for activity and growth. As observed in some studies [21, 22], the concentration of glucose is reduced in milk samples from cows with clinical mastitis. The exact reason for this change is unknown, but one possible explanation is restriction of blood flow into the infected mammary gland, resulting in a limited blood glucose supply to the secretory cells. An alternative hypothesis is that glucose is transported out of milk via a paracellular pathway, to maintain osmotic balance between the extracellular environment and the milk, where Na+ and Cl-increase in milk during mastitis [23]. In addition, the levels of some TCA cycle intermediates, such as citric acid, succinic acid, and malic acid, also increased significantly in the milk samples after treatment with LAB in this study, suggesting that the TCA cycle is upregulated during the LAB cleaning process.

Lipids, such as free fatty acids (FFA) (oleic acid, linoleic acid, palmitic acid) and glycerol are important components of milk. During mastitis, the extent of lipolysis increases and this leads to an elevated level of FFAs in milk [24]. We observed that FFAs showed a trend toward up-regulation trend from 0 to 10 d in the LAB groups. This elevation in the FFA level is interesting because even trace amounts of FFAs have exhibited inhibitory effects against *S. aureus*, a pathogen commonly associated with lactational mastitis. It is possible that the elevation of FFA is an element of the non-specific immune response to pathogen-associated lactational mastitis. It is notable that previous studies demonstrated that the potency of these compounds varies among bacterial strains [25]. Carnitine metabolism is another metabolic pathway that was up-regulated from 0 to 10 d in this study. L-carnitine is responsible for the transportation of long-chain fatty acids, such as palmitic acid, stearic acid, oleic acid, linoleic acid, and arachidonic acid from the cytosol to the mitochondrial matrix, and thus further regulating energy metabolism [26]. With the vital function of L-carnitine as a mediator of the shuttling of long-chain fatty acids into the mitochondria for β-oxidation [27, 28], the increases in L-carnitine in both the 10 d and 12 d milk samples with LAB treatment seen in this study suggests enhanced energy metabolism in the LAB groups.

Additionally, the levels of hippurate and phenylalanine, the metabolites of phenylalanine metabolism, increased over time in the groups treated with LAB. The amount of milk hippurate was significantly reduced during the course of mastitis induced by *S. agalactiae* inoculation 36h post infection [22]. We found that the hippurate concentration increased over time, reaching its highest level after 10 days of treatment with LAB. Similarly, lactose concentration was upward to reach its highest level in 10 days, but could not be detected at 12 days. A previous research showed declined concentrations of hippurate and lactose in raw milk, associated with increased SCC [29]; it revealed that the reduced concentration of lactose could serve to maintain the osmotic pressure of, and compensate for the flow of blood constituents into, the milk. Moreover, we observed elevated concentrations of phenylalanine, which is required to maintain sufficient tetrahydrobiopterin for the production of nitric oxide by isoform of nitric acid synthase, in activated macrophages and other leukocytes [30]. It has been reported that a deficiency in phenylalanine and tyrosine impairs immune responses in chickens, and that this impairment could be reversed by dietary supplementation with these compounds [31]. However, the spermine in cow milk samples in this study showed a constantly increasing trend during the LAB cleaning process from 0 to 12 d. Although spermine is essential for developing the gut and immune system of newborns, the concentration of spermine was higher in mastitis-affected milk in a previous study [32]. The protective effect of spermine against inflammation may explain this result [33]. Furthermore, we observed an increased abundance in almost all small peptides (less than or equal to 4 amino acid residues in length) in milk samples from CD and LAB groups over the time course. It has been proposed that small peptides are associated with milk somatic cells, because the milk SCC correlates with both protease release (and subsequent proteolysis activity) and the severity of mastitis [34].

Nevertheless, one limitation of this study was its relatively small sample size (*n* = 6 or 11 per group). Further experimental validation will be necessary before the current findings can be applied in farms. Moreover, our previous study observed drastic differences in milk microbial and metabolic composition among healthy, subclinical, and clinical mastitis milk samples [8, 35-37]. The microbial composition is anticipated to have a significant influence on the milk metabolome. Thus, genomic analyses will be useful for understanding the interactions between milk metabolites and milk microbes. Specific proteomics, peptidomics, and lipidomics can also be applied to analyze protein, peptide, and lipid compounds. With an integrated omics approach, the pathological and biologic mechanisms preventing mastitis by probiotic LAB could be further elucidated.

Teat dipping with effective products is critical for the prevention of SCM. Our results indicate that both the metabolites and SCC in the raw milk can be altered by the LAB and CD. Additionally, compared with the CD groups, vitamin metabolism and protein hydrolysis were increased in the LAB groups. Furthermore, teat cleaning with LAB has advantages over that done using CD, because LAB is harmless to cows and nontoxic to milk consumers. Hence, LAB teat detergents have the potential to replace chemical detergents for keeping breast healthy. In addition, there were notable differences in the metabolism of carbohydrate, energy, lipids, peptides, amino acids, and vitamins between the cleaning and non-cleaning LAB teat dipping treatments. This may be related to the therapeutic mechanism underlying SCM during LAB treatment.

## Supplemental Materials

Supplementary data for this paper are available on-line only at http://jmb.or.kr.



## Figures and Tables

**Table 1:** Significantly differing metabolites between milk samples from LAB and CD groups in different time points, positive and negative ion analysis.

Identity	Super Pathway	Sub Pathway	Fold change	*P*-value	Trend
LAB_1 VS CD_1					
L- palmitoylcarnitine	Lipid	FA metabolism	14.46	5.55E-10	Up
Dihydrolipoamide	Carbohydrate	TCA cycle	3.12	1.85E-05	Up
Tyr-cys	Peptides	-	2.14	0.00128	Up
LAB_10 VS CD_10					
Indoleacrylic acid	-	-	3.21	0.00815	Down
Tyr-pro	Peptides	-	2	0.04888	Up
Arg-ala-glu	Peptides	-	2.96	0.01326	Up
Riboflavin	Vitamin	Riboflavin metabolism	10.84	0.03781	Down
N-Acetyl-glucosamine-1-phosphate	Glycan	Lipopolysaccharide biosynthesis	2.16	0.02232	Down
Tyr-leu-asp-leu	Peptides	-	14.58	7.21E-07	Up
N-stearoyl glycine	-	-	2.37	0.01859	Down
(cAMP) Adenosine cyclic 3',5'-monophosphate	Peptides	Cationic antimicrobial peptide	3.43	0.03086	Down
Inosine	Nucleotide	Purine metabolism	2.68	0.01592	Down
N-benzoyl aspartic acid	-	-	2.09	0.01506	Up
Biotin	Vitamin	Biotin Metabolism	2.2	0.00472	Down
Propanoyl phosphate	Energy	Carbon metabolism	3.38	0.00065	Up
Lys-Trp	Peptides	-	2.29	0.00589	Up
LAB_12 VS CD_12					
Ciprofloxacin	Lipid	Bile secretion	61.41	0.04178	Down
Malic acid	Carbohydrate	TCA cycle	3.57	0.02617	Up
Cis-9-retinal	Vitamin	Retinal Metabolism	2.28	0.01671	Up
His-thr	Peptides	-	3.31	0.029	Up
dl-3-Indolelactic acid	-	-	22.45	0.02053	Up
Trp-Val	Peptides	-	2.05	0.01208	Up
Pro-phe-leu	Peptides	-	2.49	0.02791	Down

“-” = Unknown; FA = fatty acid; TCA = tricarboxylic acid cycle; Trend = the change in expression in the CD group relative to the LAB group.

**Table 2:** Significant differing metabolites between milk samples from LAB groups in different time points, positive and negative ion analysis.

Identities	Super Pathway	Sub Pathway	Fold change	*P*-value	Trend
LAB_0 VS LAB_1					
Stearic acid	Lipid	Glycerolipid metabolism	17.43	5.49E-10	Down
L-glutamate	Amino acid	Ala, Asp, Glu metabolism	17.43	2.23E-06	Down
N-hydroxytyrosine	-	-	3.92	2.44E-10	Down
L-dopaquinone	-	-	4.11	1.16E-08	Down
Cystine	Amino acid	Cys and Met metabolism	3.28	5.53E-08	Down
Carnosine	Amino acid	His metabolism	2.01	0.02299	Down
Cytidine	Nucleotide	Pyrimidine metabolism	3.38	1.52E-06	Down
Cholic acid	Lipid	Primary bile acid biosynthesis	3.66	2.36E-06	Down
Valproic acid	Lipid	Bile secretion	7.18	0.00183	Down
Erucic acid	Lipid	Biosynthesis of UFA	3.22	6.55E-06	Up
Thymidine	Nucleotide	Pyrimidine metabolism	4.23	7.57E-06	Up
His-pro	Peptides	-	2.71	0.00375	Up
Glycidyl oleate	-	-	2.45	0.00607	Up
Dobutamine	-	-	2.21	0.01824	Up
Palmitic acid	Lipid	Glycerolipid metabolism	29.49	0.02773	Up
N-Acetyl-L-cysteine	Amino acid	Glutathione metabolism	2.05	0.02134	Down
N1-Acetylspermine	-	-	2.63	0.00831	Up
Arachidic acid	Lipid	Biosynthesis of UFA	5.56	0.02365	Down
Phenylacetic acid	Amino acid	Phe metabolism	20.89	0.0049	Down
Sucralose	Carbohydrate	Carbohydrate digestion and absorption	63.46	1.90E-05	Up
Linoleic acid	Lipid	Linoleic acid metabolism	20.62	0.00657	Up
Urea	Amino acid	Urea cycle	19.26	1.40E-06	Up
Val-pro	Peptides	-	10.24	0.04079	Up
Lysophosphatidylcholine	-	-	10.02	0.01526	Up
Isoprenaline	-	-	7.12	0.01646	Up
Myristoleic acid	-	-	6.48	0.00671	Up
Citric acid	Carbohydrate	TCA cycle	5.31	0.55068	Up
Aspartate	Amino acid	Urea cycle	4.87	0.01661	Up
Spermine	Amino acid	Ala, Asp, Glu metabolism	4.73	0.02614	Up
Tocopheryl acetate	-	-	4.26	0.00115	Up
Lactic acid	Carbohydrate	Central carbon metabolism	2.01	0.02221	Up
Glucose	Carbohydrate	Glycolysis metabolism	2.26	0.00181	Up
Hippurate	Amino acid	Phenylalanine metabolism	2.26	0.00451	Up
Fructose 6-phosphate	Carbohydrate	Glycolysis metabolism	2.34	0.00215	Up
L-threonine	Amino acid	Protein metabolism	2.94	0.04657	Up
LAB_1 VS LAB_10					
Perindoprilat	-	-	2.96	0.00121	Down
His-ser-pro	Peptides	-	2.15	0.00453	Down
Carbobenzyloxy-LPhenylalanyl-L-serine	-	-	2.12	6.28E-05	Up
Butyl acetate	Amino acid	Val, Leu and Iso degradation	2.14	0.00632	Up
Oleic acid	Lipid	Glycerolipid metabolism	2.25	0.00596	Up
Lactose	Carbohydrate	Carbohydrate digestion and absorption	2.46	0.00020	Up
Linoleic acid	Lipid	Linoleic acid metabolism	2.64	0.00953	Up
Glycerol	-	-	2.67	5.05E-05	Up
Inosine	-	-	4.08	0.02420	Up
Palmitic acid	Lipid	Glycerolipid metabolism	24.59	8.00E-05	Up
pantothenic acid	Vitamin	Vitamin digestion and absorption	12.75	0.00029	Up
Deoxycytidine	Nucleotide	Pyrimidine metabolism	25.11	9.65E-07	Down
Myristic acid	-	-	3.82	4.61E-06	Up
Propyl phenylacetate	-	Microbial metabolism in diverse environments	4.08	0.00016	Up
Benzyl phenylacetate	-	Microbial metabolism in diverse environments	5.89	0.04624	Down
(2S)-2-Aminotridecanoic acid	-	-	6.81	0.04248	Up
Riboflavin	Vitamin	Riboflavin metabolism	6.25	3.60E-05	Up
Pyrazinoic acid	-	-	220.55	3.48E-09	Up
O-Phosphoserine	-	-	4.44	0.00741	Up
Succinic acid	Carbohydrate	TCA cycle	2.14	1.03E-05	Up
ESP	-	Pathogenic Escherichia coli infection	2.28	0.002	Up
Fructose 6-phosphate	Carbohydrate	Glycolysis degradation	2.4	0.00402	Up
Phenylalanine	Amino acid	Phe metabolism	2.75	0.02755	Up
Ethyl lactate	Amino acid	Cys and Met metabolism	2.88	0.01104	Up
Choline theophyllinate	-	-	3.51	0.00193	Up
Myristoleic acid	-	-	4.13	0.03023	Up
Lactose	Carbohydrate	Carbohydrate digestion and absorption	4.31	0.00156	Up
Glucose	Carbohydrate	Glycolysis metabolism	4.83	0.00055	Up
Linoleic acid	Lipid	Biosynthesis of UFA	5.64	0.00135	Up
Thr-trp	Peptides	-	6.72	0.00039	Up
Arg-pro	Peptides	-	9.28	0.03056	Up
Galactinol	Carbohydrate	Galactose metabolism	10.79	1.65E-05	Up
Leu-pro	Peptides	-	2.16	0.00353	Up
L-Carnitine	Lipid	FA degradation	45.62	0.00012	Up
Indoxyl	Amino acid	Try metabolism	83.64	0.00038	Up
LAB_10 VS LAB_12					
N-(4-Methoxybenzyl) glutamine	-	-	3.51	0.0006	Down
Myristic acid	-	-	3.71	0.02127	Down
Leu-his-lys	Lipid	-	2.04	0.00077	Up
Tyr-pro	Lipid	-	2.03	0.03285	Up
Butyl acetate	Amino acid	Val, Leu, Iso degradation	2.21	0.02878	Up
Phe-ser	Lipid	-	2.71	0.00955	Up
Erucic acid	Lipid	FA metabolism	5.12	0.0478	Up
1-Hexadecanol	Lipid	FA metabolism	4.17	0.01452	Down
Pantetheine	Vitamin	Pantothenate and CoA biosynthesis	Infinity	0.03233	Up
L-allysine	Amino acid	Biosynthesis of amino acid	2.10	0.01977	Up
Phosphoribosylamine	Amino acid	Ala, Asp, Glu metabolism	2.76	0.04867	Up
Leucylglycine	Peptides	-	7.04	0.04023	Up
N-Acetyl-5-oxonorvaline	-	-	5.04	0.01421	Up
Spermine	Amino acid	Ala, Asp, Glu metabolism	2.28	0.02663	Up
Palmidrol	-	-	4.95	0.00645	Up
Ethyl cinnamate	-	-	2.47	0.01934	Up
Ethyl hexanoate	-	-	3.02	0.04517	Up
Palmitic acid	Lipid	Glycerolipid metabolism	3.32	5.27976	Up
Fructose 6-phosphate	Carbohydrate	Glycolysis metabolism	3.72	0.00776	Up
Glucose	Carbohydrate	Glycolysis metabolism	3.48	0.00637	Up
L-carnitine	Lipid	FA degradation	92.85	0.00030	Up

“-” = Unknown; FA = fatty acid; TCA = tricarboxylic acid cycle; UFA = unsaturated fatty acid; Trend = the change in expression in the LAB_1 group relative to the LAB_0 B group, the LAB_10 group relative to the LAB_1 group, and the LAB_12 group relative to LAB_10 group.
