# Supplementary material for: Evaluation of the Efficacy of a Lactobacilli-Based Teat Detergents for the Microbiota of Cows Teats Using an Untargeted Metabolomics Approach
Source: J Microbiol Biotechnol. 2023 Sep 22;34(1):103–15. doi: 10.4014/jmb.2305.05016 (PMC10840472; doi:10.4014/jmb.2305.05016)
Supplement: Supplementary file 1 [file jmb-34-1-103-supple.pdf]

J. Microbiol. Biotechnol.
https://doi.org/10.4014/jmb.2308.08006

Supplementary Figures

Figure S1. Variables ranked by variable importance in projection (VIP), and Receiver-operator characteristic (ROC) curve for LAB_0 VS LAB_1, LAB_1 VS LAB_10, and LAB_10 VS LAB_12 in positive ion mode.

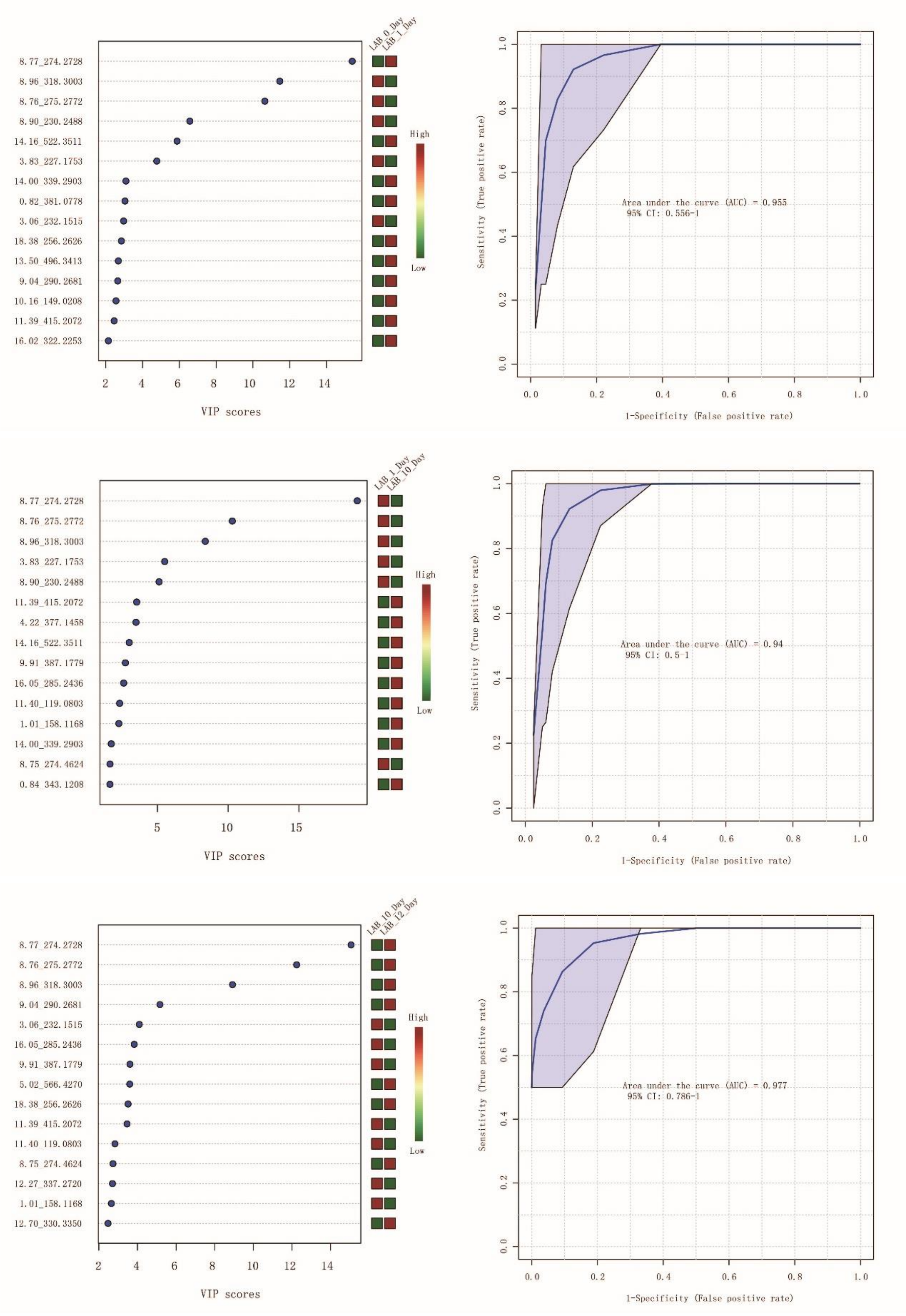

Figure S2. Variables ranked by variable importance in projection (VIP), and Receiver-operator characteristic (ROC) curve for LAB_0 VS LAB_1, LAB_1 VS LAB_10, and LAB_10 VS LAB_12 in negative ion mode.

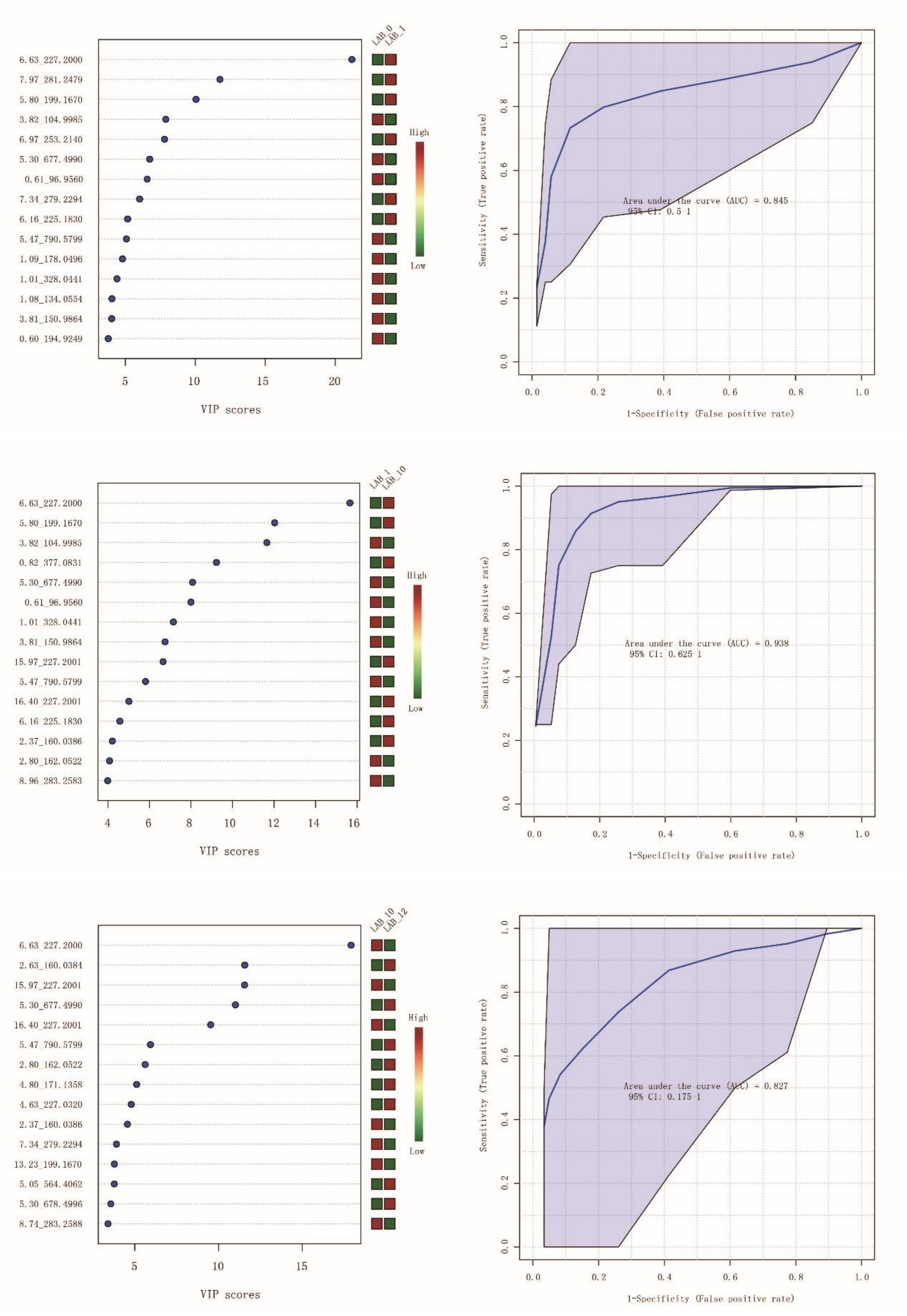

Table S1. Identification of major cow milk metabolites between LAB and CD groups from different time points by UPLC-Q-TOF MS and MS^E.

Ion Mode	RT (min)	Identities	Formula $[M+H]^+$	Exact mass	Actual mass	Mass Error (ppm)	MS Fragments $[ESI^+]$	PubChem CID
**LAB_1 VS CD_1**								
**POS**	13.3130	L-Palmitoylcarnitine	$C_{23}H_{45}NO_4$	-	400.3752	-	-	11953816
**NEG**	15.6436	Dihydrolipoamide	$C_8H_{17}NOS_2$	206.3646	206.1608	1.0	76, 191, 163, 132	663
	3.7835	Tyr-cys	$C_{12}H_{16}N_2O_4S$	-	283.0794	-	-	44631442
**LAB_10 VS CD_10**								
**POS**	3.4977	Indoleacrylic acid	$C_{11}H_9NO_2$	188.2026	188.0696	0.7	170, 143, 127, 118, 115, 91	5355219
	4.0654	Tyr-pro	$C_{14}H_{18}N_2O_4$	279.3117	279.1328	0.6	70, 106, 107, 243	15094228
	4.4479	Arg-ala-glu	$C_{14}H_{26}N_6O_6$	375.4008	375.1277	0.7	243, 198, 172	16570351
	4.2238	Riboflavin	$C_{17}H_{20}N_4O_6$	377.3718	377.1458	0.6	243, 198, 172, 105	493570
	5.1773	Tyr-leu-asp-leu	$C_{25}H_{38}N_4O_8$	-	523.2502	-	-	14154133
	6.1911	N-Stearoylglycine	$C_{20}H_{39}NO_3$	-	342.2370	-	-	4593710
**NEG**	1.0097	cAMP	$C_{10}H_{12}N_5O_6P$	328.2139	328.0441	0.5	134, 78	6076
	2.8605	Inosine	$C_{10}H_{12}N_4O_5$	267.2340	267.1214	0.4	79, 114, 267	6021
	4.7935	N-Benzoylaspartic acid	$C_{11}H_{11}NO_5$	236.2167	236.1002	0.5	177, 148, 192	95664
	0.8405	Biotin	$C_{10}H_{16}N_2O_3S$	243.3186	243.0560	1.1	78, 168, 152	171548
	2.0313	Propanoyl phosphate	$C_3H_7O_5P$	-	153.0167	-	-	1007
	2.6866	Lys-Trp	$C_{17}H_{24}N_4O_3$	-	331.1015	-	-	2193492
**LAB_12 VS CD_12**								
**POS**	0.6978	Malic acid	$C_4H_6O_5$	132.0954	132.9323	6.2	96, 276	525

	8.9568	Cis-9-retinal	$C_{20}H_{28}O$	-	283.2583	-	-	6436082
	3.7830	His-thr	$C_{10}H_{16}N_4O_4$	-	255.0865	-	-	8306814
**NEG**	2.8578	dl-3-Indolelactic acid	$C_{11}H_{11}NO_3$	204.2179	204.0639	0.8	263, 82, 107	92904
	6.8185	Trp-Val	$C_{16}H_{21}N_3O_3$	-	302.1480	-	-	7009658
	16.2453	Pro-phe-leu	$C_{20}H_{29}N_3O_4$	-	376.2525	-	-	352802

Notes: POS: positive ion mode NEG: negative ion mode; “-” means Unknown.

Table S2 Identification of major cow metabolites between LAB groups from different time points by UPLC-Q-TOF MS and MS^E.

Ion Mode	RT (min)	Identities	Formula $[M+H]^+$	Exact mass	Actual Mass	Mass Error (ppm)	MS Fragments $[ESI^+]$	PubChem CID
**LAB_0 VS LAB_1**								
**POS**	8.7367	Stearic acid	$C_{18}H_{36}O_2$	283.4852	283.2588	7.9	281, 279	5281
	0.8100	L-glutamate	$C_5H_7NO_4$	144.1054	145.1145	6.9	148, 99, 145, 71	5460299
	0.7356	N-hydroxytyrosine	$C_9H_{11}NO_4$	195.1959	195.9727	3.9	96	174052
	0.7344	L-dopaquinone	$C_9H_9NO_4$	193.1800	193.9763	4.1	96, 99	682
	2.8605	Cystine	$C_8H_{16}N_2O_4S_2$	267.3616	267.0781	1.1	88, 114	67678
	3.0698	Carnosine	$C_9H_{14}N_4O_3$	225.2325	225.1553	0.3	225, 130, 111	439224
	3.8084	Cytidine	$C_9H_{13}N_3O_5$	242.2246	242.1734	0.2	198, 241, 170	6175
	5.0446	Cholic acid	$C_{24}H_{40}O_5$	407.5793	407.2787	0.7	407, 325, 345	221493
	8.3291	Valproic acid	$C_8H_{16}O_2$	143.2194	143.1014	0.8	143, 125, 146	3121
	19.05	Erucic acid	$C_{22}H_{42}O_2$	-	321.3155	-	-	5281116
	20.72	Thymidine	$C_{10}H_{14}N_2O_5$	-	281.0516	-	-	5789
	5.91	His-pro	$C_{11}H_{16}N_4O_3$	-	546.2745	-	-	152322
	14.05	Glycidyl oleate	$C_{33}H_{38}O_6$	-	339.2896	-	-	5354568
**NEG**	6.6157	Dobutamine	$C_{18}H_{23}NO_3$	300.3881	300.2561	0.4	227, 211, 253	36811
	8.3568	Palmitic acid	$C_{16}H_{32}O_2$	255.4320	255.2290	0.8	255, 238	985
	1.3112	N-Acetyl-L-cysteine	$C_5H_9NO_3S$	162.2028	162.0519	0.9	147, 160, 134	12035
	4.8073	N1-Acetylspermine	$C_{12}H_{28}N_4O$	243.3769	243.1960	0.7	243, 225, 183	916
	10.04	Arachidic acid	$C_{20}H_{40}O_2$	-	311.2939	-	-	10467
	7.55	Phenylacetic acid	$C_8H_8O_2$	-	407.1513	-	-	999
	6.52	Sucralose	$C_{12}H_{21}N_5O_3$	-	328.1648	-	-	71485
	7.01	Linoleic acid	$C_{18}H_{32}O_2$	-	301.2155	-	-	5280450

	3.04	Urea	CH_4N_2O	-	60.0553	-	-	1176
	1.97	Val-pro	$C_{10}H_{18}N_2O_3$		235.1070			5003412
	12.40	Lysophosphatidylcholine	$C_{22}H_{46}NO_7P$	-	466.2931	-	-	5311264
	12.04	Isoprenaline	$C_{11}H_{17}NO_3$	-	421.2352	-	-	3779
	6.10	Myristoleic acid	$C_{14}H_{26}O_2$	-	225.1845	-	-	5281119
	0.55	Citric acid	$C_6H_8O_7$	-	192.1235	-	-	311
	6.34	Aspartate	$C_4H_5NO_4$	-	131.0868	-	-	5460541
	4.01	Spermine	$C_{10}H_{26}N_4$	-	239.1639	-	-	1103
	23.04	Tocopheryl acetate	$C_{31}H_{52}O_3$	-	517.3925	-	-	86472
	0.99	Lactic acid	$C_3H_6O_3$	-	135.0294	-	-	612
	9.81	Glucose	$C_6H_{12}O_6$	-	180.1559	-	-	5793
	1.08	Hippurate	$C_9H_9NO_3$	-	200.0315	-	-	464
	3.73	Fructose 6-phosphate	$C_6H_{13}O_9P$	-	260.1358	-	-	62713
	2.52	L-threonine	$C_4H_9NO_3$	-	164.0567	-	-	6288
**LAB_1 VS LAB_10**								
**POS**	5.4391	Perindoprilat	$C_{17}H_{28}N_2O_5$	341.4226	341.2416	0.5	209, 114, 96	72022
	5.2739	His-ser-pro	$C_{14}H_{21}N_5O_5$	340.3550	340.2566	0.2	132, 114, 113, 96, 79	16571789
	9.9100	Carbobenzyloxy-L-phenylalanyl-L-serine	$C_{21}H_{26}N_2OS_2$	387.5739	387.1779	1.0	105, 93	4078
	1.01	Butyl acetate	$C_6H_{12}O_2$	-	158.1176	-	-	31272
	14.05	Oleic acid	$C_{18}H_{34}O_2$	-	265.2531	-	-	445639
	0.81	Lactose	$C_{12}H_{22}O_{11}$	-	342.1163	-	-	6134
	12.64	Linoleic acid	$C_{18}H_{32}O_2$	-	263.2376	-	-	5280450
	16.07	Glycerol	$C_3H_8O_3$		92.0938			753
	1.08	Inosine	$C_{10}H_{12}N_4O_5$	-	600.1820	-	-	6021
	19.46	Palmitic acid	$C_{16}H_{32}O_2$	-	298.2744	-	-	985
	0.8159	Pantothenic acid	$C_9H_{17}NO_5$	218.2429	218.0992	0.7	71, 146, 99	6613

	3.0693	Deoxycytidine	$C_9H_{13}N_3O_4$	226.2252	226.1620	0.3	225, 130, 111	13711
**NEG**	16.3961	Myristic acid	$C_{14}H_{28}O_2$	227.3789	227.2001	0.8	227, 209	11005
	0.8020	Propyl phenylacetate	$C_{11}H_{14}O_2$	177.2356	177.0199	1.2	80, 122, 135	221641
	2.9569	Benzyl phenylacetate	$C_{15}H_{14}O_2$	225.2784	225.0639	1.0	152, 78, 225, 149	60999
	15.9736	(2S)-2-Aminotridecanoic acid	$C_{13}H_{27}NO_2$	228.3669	228.1999	0.7	227, 209	656741
	3.7785	Riboflavin	$C_{17}H_{20}N_4O_6$	376.1382	376.1286	0.03	212, 255	493570
	1.4375	Pyrazinoic acid	$C_5H_4N_2O_2$	123.0975	123.0402	0.5	123, 108, 105	1047
	1.4944	O-phosphoserine	$C_3H_8NO_6P$	184.0645	184.0974	0.2	138, 184, 74	68841
	22.04	Succinic acid	$C_4H_6O_4$	-	118.0880	-	-	1110
	0.95	ESP	$C_6H_{13}O_9P$	-	259.0213	-	-	82644
	3.73	Fructose 6-phosphate	$C_{12}H_{23}O_{14}P$	-	403.0657	-	-	62713
	1.26	Phenylalanine	$C_9H_{11}NO_2$	-	146.0604	-	-	6140
	3.34	Ethyl lactate	$C_5H_{10}O_3$	-	281.1256	-	-	7344
	6.85	Choline theophyllinate	$C_{12}H_{21}N_5O_3$	-	328.1652	-	-	656652
	6.10	Myristoleic acid	$C_{14}H_{26}O_2$	-	225.1845	-	-	5281119
	9.81	Glucose	$C_6H_{12}O_6$	-	180.1559	-	-	5793
	22.38	Linoleic acid	$C_{18}H_{32}O_2$	-	559.4689	-	-	5280450
	2.81	Thr-trp	$C_{15}H_{19}N_3O_4$	-	304.1312	-	-	71728368
	2.30	Arg-pro	$C_{11}H_{21}N_5O_3$	-	292.1384	-	-	332964
	24.68	Galactinol	$C_{12}H_{22}O_{11}$	-	363.0895	-	-	11727586
	2.16	Leu-pro	$C_{11}H_{20}N_2O_3$	-	227.1382	-	-	3977067
	1.67	L-Carnitine	$C_7H_{15}NO_3$	-	182.0806	-	-	10917
	1.77	Indoxyl	C_8H_7NO	-	132.0444	-	-	50591
**LAB_10 VS LAB_12**								
**POS**	10.5767	N-(4-Methoxybenzyl) glutamine	$C_{13}H_{18}N_2O_4$	267.3010	267.1203	0.7	267, 159, 107, 81	21600975
	10.5969	Myristic acid	$C_{14}H_{28}O_2$	229.3789	229.1796	0.9	103, 85, 71	11005

	11.3588	Leu-his-lys	$C_{18}H_{32}N_6O_4$	397.4924	397.1981	0.7	136, 120	10095662
	4.06	Tyr-pro	$C_{14}H_{18}N_2O_4$	-	279.1344	-	-	9795637
	1.01	Butyl acetate	$C_6H_{12}O_2$	-	158.1176	-	-	31272
	20.74	Phe-ser	$C_{12}H_{16}N_2O_4$	-	297.0833	-	-	193508
	13.77	Erucic acid	$C_{22}H_{42}O_2$	-	402.3373	-	-	5281116
**NEG**	17.2968	1-Hexadecanol	$C_{16}H_{34}O$	241.4485	241.2150	1.0	241	2682
	6.9193	Pantetheine	$C_{11}H_{22}N_2O_4S$	277.3763	277.2123	0.6	279, 261	439322
	5.9006	L-allysine	$C_6H_{11}NO_3$	144.1644	144.0414	0.9	144, 115	160603
	4.6190	Phosphoribosylamine	$C_5H_{12}NO_7P$	228.1250	228.0353	0.4	171, 227, 143	3082052
	2.7471	Leucylglycine	$C_8H_{16}N_2O_3$	187.2242	187.1113	0.6	141, 113	79070
	4.7946	N-Acetyl-5-oxonorvaline	$C_7H_{11}NO_4$	172.1665	172.1409	0.1	171, 153	65784
	5.4069	Spermine	$C_{10}H_{26}N_4$	201.3402	201.1098	1.1	201, 78	1103
	6.1604	Palmidrol	$C_{18}H_{37}NO_2$	298.4919	298.2469	0.8	297, 152, 78	4671
	9.83	Ethyl cinnamate	$C_{11}H_{12}O_2$	-	175.0749	-	-	637758
	5.24	Ethyl hexanoate	$C_8H_{16}O_2$	-	287.2213	-	-	31265
	5.28	Palmitic acid	$C_{16}H_{32}O_2$	-	277.2140	-	-	985
	3.73	Fructose 6-phosphate	$C_6H_{13}O_9P$	-	260.1358	-	-	62713
	9.81	Glucose	$C_6H_{12}O_6$	-	180.1559	-	-	5793
	1.67	L-carnitine	$C_7H_{15}NO_3$	-	182.0806	-	-	10917
	23.11	Lactose	$C_{12}H_{22}O_{11}$		363.0894			6134

Notes: POS: positive ion mode NEG: negative ion mode; “-” means Unknown.
